# Characterization of Shiga toxin-producing *Escherichia coli* O130:H11 and O178:H19 isolated from dairy cows

**DOI:** 10.3389/fcimb.2013.00009

**Published:** 2013-03-08

**Authors:** Daniel Fernández, Alejandra Krüger, Rosana Polifroni, Ana V. Bustamante, A. Mariel Sanso, Analía I. Etcheverría, Paula M. A. Lucchesi, Alberto E. Parma, Nora L. Padola

**Affiliations:** Laboratorio de Inmunoquímica y Biotecnología, Facultad Ciencias Veterinarias, Centro de Investigaciones Veterinarias Tandil-Consejo Nacional de Investigaciones Científicas y Técnicas-Comisión de Investigaciones Científicas de la Provincia de Buenos Aires (CIVETAN-CONICET-CICPBA), Universidad Nacional del Centro de la Provincia de Buenos AiresTandil, Argentina

**Keywords:** STEC, dairy cattle, MLVA, Shiga toxin

## Abstract

Shiga toxin-producing *E. coli* (STEC) are isolated from human patients with bloody diarrhea, hemorrhagic colitis (HC), and hemolytic uremic syndrome (HUS). In the last years, the infections with non-O157 serotypes are increasing their frequency of association with human disease. STEC produce Shiga toxin (Stx) and other virulence factors that could contribute to human pathogenesis. Cattle are the main reservoir and the transmission to humans is through the consumption of undercooked meat, non-pasteurized dairy products, and vegetables or water contaminated with feces. We have previously determined that O130:H11 and O178:H19 serotypes were the most prevalent in dairy cows from Argentina. In the present study, 37 and 25 STEC isolates from dairy cows belonging to O130:H11 and O178:H19 serotypes, respectively, were characterized regarding to their cytotoxicity on Vero cells, *stx* subtypes, presence of *sab* and typing by multiple-locus variable-number tandem repeat analysis (MLVA). All strains demonstrated a cytotoxic effect, and in O130:H11 isolates, *stx*2_EDL933_ was the predominant subtype. In O178:H19 isolates the main *stx2* subtype was *stx*2_vha_. The *sab* gene was detected in 65 and 24% of the isolates belonging to O130:H11 and O178:H19, respectively. Only one MLVA profile was identified among the O130:H11 isolates meanwhile 10 MLVA profiles were detected among the O178:H19 isolates which were grouped in two main clusters. In conclusion, our data show that O130:H11 and O178:H19 STEC isolates encode virulence factors associated with severe human disease and both serotypes should be considered for routinely testing. Our subtyping experiments showed that isolates could be distinguished based on the *stx*_2_ subtype and the presence/absence of *sab* gene, and for isolates belonging to O178:H19, also when the MLVA type was considered. However, MLVA subtyping of O130:H11 isolates will require the development of more specific markers.

## Introduction

Shiga toxin-producing *E. coli* (STEC) cause bloody diarrhea, hemorrhagic colitis (HC) and hemolytic uremic syndrome (HUS) in humans (Pearce et al., 2004; Giugno et al., 2007). Most outbreaks have been attributed to O157:H7 serotype (Mora et al., 2004) but infections with non-O157 serotypes are also being frequently associated with HC and HUS (Bettelheim, 2007). In several countries STEC O157:H7 have been frequently isolated from cattle but several studies in Argentina have detected mainly non-O157:H7 serotypes (Meichtri et al., 2004; Padola et al., 2004; Fernández et al., 2010). Cattle are the main reservoir of STEC and the transmission to humans occurs through the consumption of undercooked meat, non-pasteurized dairy products, and vegetables or water contaminated with feces (Hussein and Sakuma, 2005). Direct contact with cattle and dairy farm environment has been reported also as a possible source for STEC human transmission (Oliver et al., 2005).

The main virulence factor of STEC is the production of Shiga toxins (Stx1 and Stx2) (Paton and Paton, 1998; Gyles, 2007). Stx1 group includes few subtypes, while the Stx2 is a more heterogeneous group and comprises an expanding number of subtypes (such as Stx2EDL933, Stx2vha, Stx2vhb, Stx2O118, Stx2dact, Stx2e, Stx2f, and Stx2g). Stx subtypes differ in their degree of association with HC and HUS cases, being Stx2O118 (formerly identified as Stx2d-Ount), Stx2e, Stx2f, and Stx2g not frequently associated with severe human disease (Friedrich et al., 2002; Karch et al., 2005; Prager et al., 2009, 2011). Other virulence factors that could contribute to the pathogenesis are intimin, encoded by the *eae* gene and responsible for the intimate attachment of STEC to intestinal epithelial cells, an enterohaemolysin (EhxA), an autoagglutinating adhesin (Saa) and a novel STEC autotransporter (Sab) described for first time in a *saa*-positive O113:H21 strain, which participates in adhesion and biofilm formation (Herold et al., 2009). The *ehxA*, *saa*, and *sab* genes are located in a megaplasmid (Paton and Paton, 1998; Paton et al., 2001; Herold et al., 2009).

In Argentina, O130:H11 and O178:H19 were the most prevalent serotypes isolated from dairy cows (Fernández et al., 2010) and were also identified by Masana et al. (2011) in beef abattoirs and by López et al. (2012) in feedlot cattle. Both serotypes have been isolated from HC and HUS cases in several countries and have been found among human STEC isolates received between 2000–2010 by the CDC National *E. coli* Reference Laboratory (Blanco et al., 2004; Fremaux et al., 2006; Giugno et al., 2007).

In the present study, we further characterized O130:H11 and O178:H19 STEC isolated by Fernández et al. (2010) from dairy farms regarding their cytotoxicity on Vero cells, *stx* subtypes, presence of *sab* gene and typing by multiple-locus variable-number tandem repeat analysis (MLVA), in order to evaluate the genetic diversity of isolates belonging to these serotypes which are prevalent in dairy cattle.

## Materials and methods

### Bacterial strains

The bacterial strains used in this study were 37 STEC O130:H11 and 25 STEC O178:H19 isolated from dairy cows in five farms (named A, B, C, D, and E) from Argentina (Fernández et al., 2010).

### Cytotoxic activity on vero cells

The cytotoxicity of the isolates was evaluated by Vero cells assay. Briefly, each strain was cultured overnight into 25 ml of Microbiological broth (No. 3, Merck) and was centrifuged 120× g (10 min at 4°C) and the supernatant was centrifuged again 17,228× g (10 min at 4°C) and identified as S1. The cell pellet was washed with PBS, resuspended in 3 ml of polymyxin sulfate (0.1 mg/ml) and incubated 30 min. Polymyxin B-treated cultures were centrifuged at 120× g (10 min at 4°C). The supernatant was centrifuged at 17,228× g, 10 min at 4°C, and was identified as S2. Fifty and 25 μl of each one S1 and S2 were inoculated in each one of the 96-well-plates containing 4 × 10^4^ freshly trypsinized Vero cells and were incubated 48 h at 37°C in a 5% CO_2_ atmosphere. The cell monolayers were fixed with 10% (v/v) formaldehyde and then stained with 0.2% (w/v) crystal violet in phosphate-buffered saline solution. *E. coli* EDL933 strain was used as positive control and a strain *stx* positive without cytotoxic effect as negative control (*E. coli* serotype O15:H21). Wells having 50% or greater cytotoxicity, compared to a standard control well were considered positive.

### *stx* subtyping

The strategy to detect *stx*_2_ subtypes was similar to that previously described by Krüger et al. (2011). Briefly, all *stx*_2_-positive STEC were subjected to PCR with the primer pair VT2-c/VT2-d, and amplification products were independently digested with restriction endonucleases *Hae*III, *Rsa*I, and *Nci*I to detect *stx*_2EDL933_, *stx*_2vha_, *stx*_2vhb_, *stx*_2g_, and *stx*_2NV206_ (Tyler et al., 1991; Bertin et al., 2001; Krüger et al., 2007). All isolates were also evaluated with the VT2-cm/VT2-f primer set (Pierard et al., 1998) specific for *stx*_2*O*118_ (first termed *stx*_2d_ by Piérard and renamed *stx*_2*O*118_ as proposed by Scheutz and Strockbine, 2005). The strains used as positive controls for each subtype and the references corresponding to the primers are detailed in Krüger et al. (2011).

### *sab* gene

The detection of the *sab* gene was performed by PCR using the primers described by Herold et al. (2009) and the following amplification conditions: initial cycle of 94°C for 120 s, 30 cycles with denaturation step (94°C, 30 s), annealing step (54°C, 30 s) and extension step (68°C, 30 s), and a 60 s cycle at 72°C. STEC O20:H19 was used as positive control and *Salmonella spp, Staphylococcus aureus*, and *Pseudomonas aeruginosa* as negative controls.

### MLVA assay

We performed an MLVA assay that previously showed a high level of discrimination among STEC isolates belonging to different non-O157:H7 serotypes (Schimmer et al., 2008; Bustamante et al., 2010; Franci et al., 2011). The seven VNTR loci studied in this assay were analyzed as described by Bustamante et al. (2010). Representative alleles were sequenced with an ABI PRISM 3730XL genetic analyzer (Macrogen, Korea). The dendrogram was constructed using the UPGMA clustering method implemented by START Vs. 1.0.5 software (Joley et al., 2001). The alleles were indicated in a string order CVN001-CVN002-CVN003-CVN004-CVN007-CVN014-CVN015, named according to the number of tandem repeat sequences. If no amplification product was detected, the allele was designated with an arbitrary number (30).

In all PCR assays, Inbio-Highway (Argentina) DNA polymerase was used.

## Results and discussion

Using Vero cell assay, the S1 and S2 supernatants of all isolates from both serotypes demonstrated cytotoxic effect after 48 h post-inoculation on Vero cells.

Among 36 *stx*_2_-positive O130:H11 isolates, *stx*_2EDL933_ was the predominant subtype (81%), and the other subtype present was *stx*_2vhb_ (Table 1). Only three isolates harbored both subtypes.

**Table 1:** **Origin and virulence genotypes of O130:H11 isolates**.

**Strain number**	**Farm**	Virulence genotype^*^	***sab***	***stx*_2_ subtype**
1	A	*stx*_1_*-ehxA-saa*	−	*–*
2	A	*stx*_1_−stx_2_*-ehxA-saa*	−	*stx*_2vhb_
3	A	*stx*_1_−stx_2_*-ehxA-saa*	−	*stx*_2vhb_
4	A	*stx*_1_−stx_2_*-ehxA-saa*	−	*stx*_2vhb_
5	A	*stx*_1_−stx_2_*-ehxA-saa*	−	*stx*_2EDL933_
6	A	*stx*_1_−stx_2_*-ehxA-saa*	−	*stx*_2EDL933_
7	A	*stx*_1_−stx_2_*-ehxA-saa*	−	*stx*_2EDL933_
8	B	*stx*_1_−stx_2_*-ehxA-saa*	−	*stx*_2EDL933_ *stx*_2vhb_
9	B	*stx*_1_−stx_2_*-ehxA-saa*	−	*stx*_2EDL933_ *stx*_2vhb_
10	B	*stx*_1_−stx_2_*-ehxA-saa*	+	*stx*_2EDL933_
11	C	*stx*_1_−stx_2_*-ehxA-saa*	+	*stx*_2EDL933_
12	C	*stx*_1_−stx_2_*-ehxA-saa*	+	*stx*_2EDL933_
13	C	*stx*_1_−stx_2_*-ehxA-saa*	+	*stx*_2EDL933_
14	C	*stx*_1_−stx_2_*-ehxA-saa*	+	*stx*_2EDL933_
15	C	*stx*_1_−stx_2_*-ehxA-saa*	+	*stx*_2EDL933_
16	C	*stx*_1_−stx_2_*-ehxA-saa*	+	*stx*_2EDL933_
17	C	*stx*_1_−stx_2_*-ehxA-saa*	+	*stx*_2EDL933_
18	C	*stx*_1_−stx_2_*-ehxA-saa*	+	*stx*_2EDL933_
19	C	*stx*_1_−stx_2_*-ehxA-saa*	+	*stx*_2EDL933_
20	C	*stx*_1_−stx_2_*-ehxA-saa*	+	*stx*_2EDL933_
21	C	*stx*_1_−stx_2_*-ehxA-saa*	+	*stx*_2EDL933_
22	D	*stx*_1_−stx_2_*-ehxA-saa*	+	*stx*_2EDL933_ *stx*_2vhb_
23	D	*stx*_1_−stx_2_*-ehxA-saa*	−	*stx*_2EDL933_
24	D	*stx*_1_−stx_2_*-ehxA-saa*	+	*stx*_2EDL933_
25	D	*stx*_1_−stx_2_*-ehxA-saa*	+	*stx*_2EDL933_
26	D	*stx*_1_−stx_2_*-ehxA-saa*	−	*stx*_2vhb_
27	D	*stx*_1_−stx_2_*-ehxA-saa*	+	*stx*_2vhb_
28	D	*stx*_1_−stx_2_*-ehxA-saa*	+	*stx*_2EDL933_
29	D	*stx*_1_−stx_2_*-ehxA-saa*	+	*stx*_2EDL933_
30	D	*stx*_1_−stx_2_*-ehxA-saa*	+	*stx*_2EDL933_
31	D	*stx*_1_−stx_2_*-ehxA-saa*	−	*stx*_2vhb_
32	D	*stx*_1_−stx_2_*-ehxA-saa*	+	*stx*_2EDL933_
33	D	*stx*_1_−stx_2_*-ehxA-saa*	+	*stx*_2EDL933_
34	D	*stx*_1_−stx_2_*-ehxA-saa*	+	*stx*_2EDL933_
35	D	*stx*_1_−stx_2_*-ehxA-saa*	−	*stx*_2EDL933_
36	D	*stx*_1_−stx_2_*-ehxA-saa*	+	*stx*_2EDL933_
37	E	*stx*_1_−stx_2_*-ehxA-saa*	+	*stx*_2EDL933_

* *Previously determined (Fernández et al., 2010)*.

The most frequent *stx*_2_ subtype among O178:H19 isolates was *stx*_2vha_ (72%), while *stx*_2EDL933_ and *stx*_2vhb_ subtypes were found less frequently (20 and 8%, respectively) and no isolates harboring more than one *stx*_2_ subtype were found.

It is interesting to note that the *stx*_2EDL933_-positive strains, belonging to either O130:H11 or O178:H19 serotypes, (Tables 1 and 2) corresponded mainly to isolates harboring the profile *stx*_1_−*stx*_2_-*ehxA*-*saa*.

**Table 2:** **Origin and characterization of O178:H19 isolates**.

**Strain number**	**Farm**	Virulence genotype^*^	***sab***	***stx*_2_ subtype**	**MLVA profile**
1	A	*stx*_2_	−	*stx*_2vha_	I_1_
2	A	*stx*_2_	−	*stx*_2vha_	I_1_
3	A	*stx*_2_	−	*stx*_2vha_	I_1_
4	A	*stx*_2_	−	*stx*_2vha_	I_1_
5	A	*stx*_2_	−	*stx*_2vha_	I_2_
6	C	*stx*_2_	−	*stx*_2vha_	I_3_
7	C	*stx*_2_	−	*stx*_2vha_	I_5_
8	D	*stx*_2_	−	*stx*_2vha_	I_1_
9	D	*stx*_2_	+	*stx*_2vhb_	II_2_
10	E	*stx*_2_	−	*stx*_2vha_	I_1_
11	E	*stx*_2_	+	*stx*_2EDL933_	II_4_
12	E	*stx*_2_	−	*stx*_2vhb_	I_4_
13	E	*stx*_2_	−	*stx*_2vha_	I_2_
14	E	*stx*_2_	−	*stx*_2vha_	I_1_
15	B	*stx*_2_	−	*stx*_2vha_	I_3_
16	A	*stx*_2_	−	*stx*_2vha_	I_2_
17	C	*stx*_1_−stx_2_*-ehxA-saa*	+	*stx*_2EDL933_	II_3_
18	C	*stx*_1_−stx_2_*-ehxA-saa*	+	*stx*_2EDL933_	II_5_
19	D	*stx*_2_	−	*stx*_2vha_	I_2_
20	D	*stx*_1_−stx_2_*-ehxA-saa*	+	*stx*_2EDL933_	II_2_
21	D	*stx*_2_*-ehxA-saa*	−	*stx*_2vha_	I_2_
22	D	*stx*_2_	−	*stx*_2vha_	I_1_
23	E	*stx*_1_−stx_2_*-ehxA-saa*	+	*stx*_2EDL933_	II_1_
24	C	*stx*_2_	−	*stx*_2vha_	I_1_
25	C	*stx*_2_	−	*stx*_2vha_	I_1_

* *Previously determined (Fernández et al., 2010)*.

The subtypes found in this work have been reported as the predominant *sxt*_2_-subtypes in bovine STEC strains in Argentina and other countries (Bertin et al., 2001; Brett et al., 2003; Meichtri et al., 2004; Galli et al., 2010; Krüger et al., 2011) and have been associated with the development of HC and HUS (Friedrich et al., 2002; Persson et al., 2007). In a study performed by Masana et al. (2011) O130:H11 and O178:H19 were also among the most prevalent serotypes found in carcasses and bovine feces sampled at abattoirs in Argentina. In that study, O130:H11 isolates presented the same virulence genotypes (in regard to the presence of *stx*_1_, *stx*_2_ subtypes, *ehxA* and *saa*) as the ones detected in the present report. Regarding to O178:H19, some virulence genotypes (*stx*_2vha_; *stx*_1_−stx_2EDL933_-*ehxA*-*saa*; *stx*_2vhb_) found by Masana et al. (2011) were detected also in the present study, but there were other profiles (*stx*_2NT_; *stx*_2EDL933_−*stx*_2vha_) not shared between these studies.

The gene encoding Sab, a protein which mediates biofilm formation and promotes intestinal adherence, was detected in 65% of the isolates belonging to O130:H11. This study is the first, to our knowledge, to describe O130:H11 as a serotype carrying *sab*. In O178:H19 isolates *sab* was detected in 24% of the isolates (Table 2). Buvens et al. (2010) did not detect *sab* in a STEC O178:H19 strain isolated from HUS. All *sab*-positive STEC strains identified to date were also positive for *ehx* as well as *saa*, all genes located in a megaplasmid, noteworthy, in the present study some of the O178:H19 isolates were *sab*-positive but negative for *ehx*A and *saa*.

Most of the MLVA loci could be amplified, although there were differences between serotypes. To our knowledge this is the first time that STEC O130:H11 is typed by MLVA and notably, only one MLVA profile (5-2-30-9-8-30-6) was detected among these isolates. We have used this MLVA assay to subtype several isolates belonging to different non-O157:H7 serotypes and we found a high level of discrimination (Bustamante et al., 2010; Franci et al., 2011). Other authors have also applied this protocol to successfully resolve outbreaks due to a non-O157 strain (Schimmer et al., 2008). In our experience, this is the first time that all isolates from a same serotype and different origin present a unique MLVA profile. The lack of diversity found in this serotype would indicate that the chosen VNTR loci are not variable enough for typing O130:H11 strains since they did show variability in relation with the presence/absence of *sab* and also with the *stx2* subtype present. Therefore, there is a need to identify VNTR loci that are variable among STEC strains belonging to this serotype.

**Figure 1:**
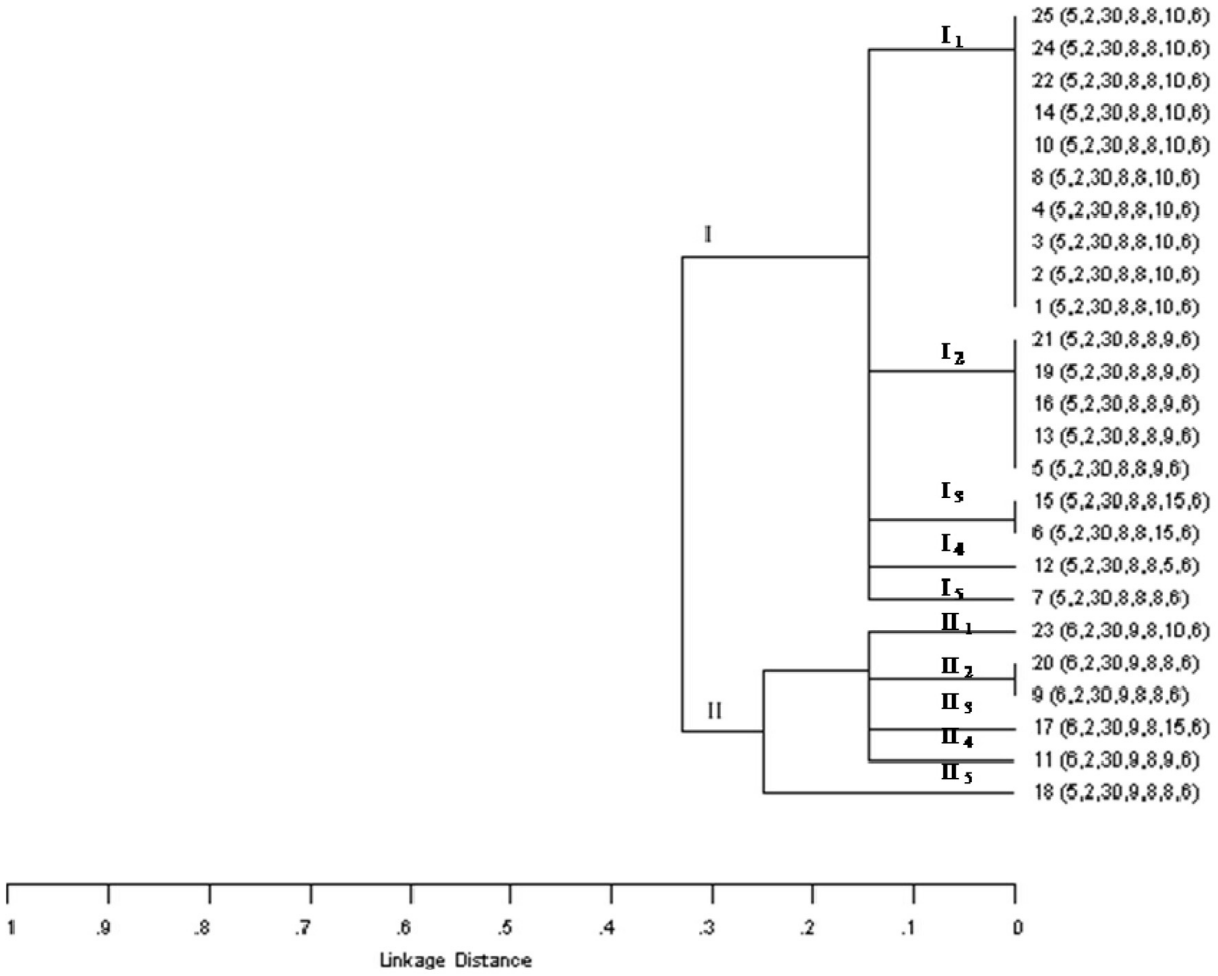
**Dendrogram based on MLVA profiles of STEC O178:H19 isolated from dairy cows in Argentina.** Order of the allele string: CVN001-CVN002-CVN003-CVN004-CVN007-CVN014-CVN015.

On the other hand, among the 25 O178:H19 isolates, 10 MLVA profiles were detected, which were grouped in two main clusters (Figure 1). A relationship could not be found with regard to MLVA profiles and farm origin (Table 2). Cluster I included isolates from all the farms, and cluster II, isolates from dairy farms C, D, and E. A high variability was found among isolates from farms C and E, detecting in each farm 5 MLVA profiles among 6 isolates (Table 2). All isolates belonging to clade I, were *sab*-negative and, with the exception of isolate 12, they presented the subtype *stx*_2vha_ (Table 2). Clade II was the most variable, presenting five different profiles among six isolates. Moreover, isolates 9 and 20 shared the MLVA profile but not their virulence profile. Within this clade, all the isolates were *sab*-positive and carried *stx*_2EDL933_, with the exception of isolate 9 (positive for *sab* but negative for that *stx*_2_ subtype) (Table 2). Although a relationship between the MLVA profile and the *stx*_2_ subtype is not expected, with the exception of isolates from a same clone, all *stx*_2vha_-positive isolates belonged to cluster I and all *stx*_EDL933_-positive isolates, to cluster II. Regarding isolates carrying *stx*_2vhb_, one belonged to cluster I and the other to cluster II. Noteworthy, all the MLVA profiles present in these isolates were quite different from the ones detected previously in STEC O178:H19 isolated from minced meat of the same geographic region (Franci et al., 2011). Taking into account all these results, a high genetic variability was evidenced among isolates belonging to this serotype. Our results showed different STEC O178:H19 clonal lineages and determined that some clones may be present in more than one farm.

## Conclusion

The data suggest differences in the genetic variability for the two serotypes. It could be assessed when the *stx*_2_ subtype and the presence/absence of *sab* gene were taken into account, and for isolates belonging to O178:H19, also when the MLVA type was considered. The MLVA typing assay chosen seems not suitable for detecting genetic differences among O130:H11 STEC isolates, and further loci need to be analyzed.

STEC non-O157 serotypes are nowadays frequently associated with outbreaks and sporadic cases of HUS and particularly, O130:H11 and O178:H19 STEC have been isolated from human patients. In our study isolates from dairy cows belonging to these serotypes possess virulence characteristics associated with the development of severe disease in humans and it would be desirable to consider them in the group of serotypes routinely investigated.

### Conflict of interest statement

The authors declare that the research was conducted in the absence of any commercial or financial relationships that could be construed as a potential conflict of interest.
